# HPV-Induced Field Cancerisation: Transformation of Adult Tissue Stem Cell Into Cancer Stem Cell

**DOI:** 10.3389/fmicb.2018.00546

**Published:** 2018-03-26

**Authors:** Carlotta Olivero, Simone Lanfredini, Cinzia Borgogna, Marisa Gariglio, Girish K. Patel

**Affiliations:** ^1^Virology Unit, Department of Translational Medicine, Novara Medical School, University of Eastern Piedmont, Novara, Italy; ^2^European Cancer Stem Cell Research Institute, Cardiff School of Biosciences, Cardiff University, Cardiff, United Kingdom

**Keywords:** HPV, field cancerization, adult tissue stem cells, skin cancer stem cells, squamous cell carcinoma

## Abstract

Field cancerisation was originally described as a basis for multiple head and neck squamous cell carcinoma (HNSCC) and is a pre-malignant phenomenon that is frequently attributable to oncogenic human papillomavirus (HPV) infection. Our work on β-HPV-induced cutaneous squamous cell carcinomas identified a novel Lrig1+ hair follicle junctional zone keratinocyte stem cell population as the basis for field cancerisation. Herein, we describe the ability for HPV to infect adult tissue stem cells in order to establish persistent infection and induce their proliferation and displacement resulting in field cancerisation. By review of the HPV literature, we reveal how this mechanism is conserved as the basis of field cancerisation across many tissues. New insights have identified the capacity for HPV early region genes to dysregulate adult tissue stem cell self-renewal pathways ensuring that the expanded population preserve its stem cell characteristics beyond the stem cell niche. HPV-infected cells acquire additional transforming mutations that can give rise to intraepithelial neoplasia (IEN), from environmental factors such as sunlight or tobacco induced mutations in skin and oral cavity, respectively. With establishment of IEN, HPV viral replication is sacrificed with loss of the episome, and the tissue is predisposed to multiple cancer stem cell-driven carcinomas.

## Introduction

Human papillomavirus (HPV) infection is associated with oropharyngeal and anogenital cancers in both men and women. Approximately 90% of all cervical cancers are attributed to high-risk alpha-genus HPV (α-HPV) infections, also ∼60% of squamous cell carcinomas (SCC) of the vulva, vagina, anus and penis are due to infection of α-HPV (Crow, 2012). HPV infection is considered to be responsible for the rise in head and neck squamous cell carcinoma (HNSCC), particularly in cancers of the oropharynx and base of tongue (Marur et al., 2010; Leemans et al., 2011). Cutaneous HPVs, which are clustered in the evolutionarily distinct β-genus, have been also associated with the development of cutaneous SCC, especially in the immunosuppressed setting (Howley and Pfister, 2015; Quint et al., 2015).

Sequential genetic and epigenetic changes occur over several years and provide the transformational basis for both intraepithelial neoplasia (IEN) and ensuing epithelial cancers (carcinoma). The proportion of transformed cells within IEN can be graded and used to define the risk of invasive disease (FIGO Committee on Gynecologic Oncology, 2014). Progression to invasive carcinoma from IEN can take many years and there is often evidence of IEN at the excised tumor margins (Mao et al., 1996; Scholes et al., 1998).

As an entity, carcinoma account for over 70% of all malignancies and over 70% of all cancer mortality (Cancer Research UK, 2017^1^), hence the American Association for Cancer Research Task Force on the treatment and prevention of IEN recognizes the importance of early treatment to prevent invasive disease (O’Shaughnessy et al., 2002). Intriguingly, IEN can spontaneously regress. Although more often, IEN will progress to invasive malignancies (Dakubo et al., 2007). In epithelia susceptible to HPV infection, HPV early genes can cause IEN, notably E6 and E7 expression, and is therefore a mechanistic link to cancer, as such it represents a target for cancer prevention and is therefore the basis of this review.

## Field Cancerisation

Field cancerization, as a concept, was coined by Slaughter et al. (1953) to explain the occurrence of multiple foci of HNSCC. In all 783 HNSCC cases studied, the authors noted IEN at the peripheral margins of the resected primary malignancy. Where the tumor depth was less than 1 cm, they identified a second primary SCC focus in 43 of 88 cases. It is now clear that establishment of a premalignant epithelium, field cancerisation, is the basis for HNSCC, skin and cervical SCC.

**FIGURE 1:**
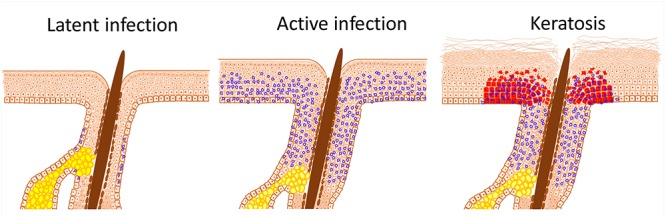
HPV infection induced stem cell expansion. Latent HPV infection persists within epithelial stem cells and, in the case of β-HPV, within hair follicle keratinocyte stem cells; characterized herein by the presence of viral episomes (blue circles). Active replication, as occurs after immunosuppression or trauma, results in proliferation of stem cells beyond the niche; in the case of the skin into the interfollicular epidermis (IFE). Expansion and displacement of keratinocyte stem cells into the IFE renders them susceptible to UV induced transformation, resulting in field cancerisation. The archetypal initial lesion is clonal p53 mutation expression, within actinic keratoses (red cells).

**FIGURE 2:**
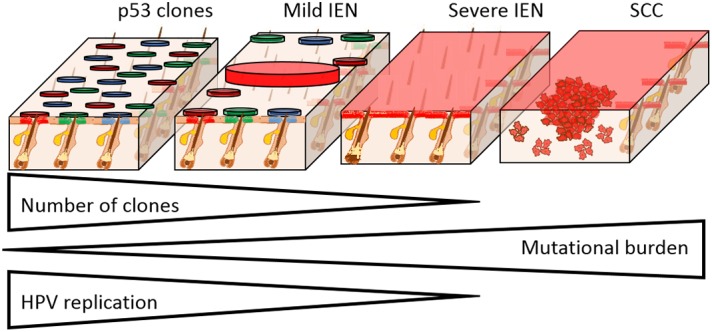
Field cancerisation in the setting of HPV infection. The progression from IEN to SCC formation is characterized by the clonal selection, acquisition of mutations, and loss of HPV replication. Skin is used here as a HPV susceptible tissue, as we have previously shown that clonal expansion occurs from the hair follicle junctional zone. In mild IEN there are many clones, which in the case of p53 mutation susceptible tissues, may harbor individual distinct mutations as shown (circles with different colors). Progression of IEN is characterized by the selection and expansion of individual clones that have gained a proliferative advantage from additional mutations, culminating in severe IEN with full thickness epidermal dysplasia that is genetically uniform. With increasing mutational load, epidermal dysplasia increases and HPV episomes are lost. In the case of β-HPV types, the entire viral infection is lost, which accounts for the “hit and run” mechanism of transformation. From within the severe IEN, foci of SCC develop as shown, resulting in invasion into the underlying tissue.

Within field cancerisation, cells harbor a substantial number of mutations including those within known tumor suppressor genes, most frequently within the p53 gene (Bartkova et al., 1995; Ortiz et al., 2001; van Houten et al., 2002; Elgazzar et al., 2005; Merrick et al., 2006; Hu et al., 2012). In many tissues, the presence of mutant p53 clones is widely accepted as the hallmark of field cancerisation (**Figure 1**) (El-Naggar et al., 1995; Franklin et al., 1997). However, in cervical IEN, p53 is less frequently mutated (Akasofu and Oda, 1995). Within the early stage of field cancerisation there are multiple clones, but with increasing dysplasia severe field cancerisation becomes monoclonal (**Figure 2**) (Chung et al., 1995; Enomoto et al., 1997; Tate et al., 1997). Therefore, field cancerisation at its outset is polyclonal, implying that multiple cell lineages contribute to its occurrence as observed in active HPV infection.

Increasing mutational burden and greater dysplasia result in clonal selection, with a tendency toward mono-clonality (**Figure 2**). Clonal selection and expansion may result in a single clone in continuous epithelia (skin, oral and cervical tissues) or multiple clones in discontinuous epithelia (breast and lung) (Prevo et al., 1999; Simon et al., 2001; Larson et al., 2002; Tabor et al., 2002; Smeds et al., 2005). Within continuous epithelia, wherein HPV infection occurs, field cancerisation and ensuing cancers exhibit common epigenetic gene silencing, chromosomal anomalies, loss of heterozygosity, single nucleotide polymorphism, mutations, changes in mitochondrial genome, and altered gene expression (transcripts and proteins) (Ha et al., 2002; Tabor et al., 2004; Shen et al., 2005; Sui et al., 2006). Hence, severe IEN (part of the field cancerisation spectrum) that gives rise to multiple cancer, has limited numbers of clones.

The ensuing SCC that arise within continuous stratified squamous epithelia are clonal with respect to the underlying field cancerisation and severe IEN (**Figure 2**) (Sheu et al., 1995; Kim et al., 1996; Enomoto et al., 1997; Tate et al., 1997). However, the proliferative explosion of SCC cells results in multiple evolving clones, from acquisition of new mutations, which similarly undergo Darwinian evolutionary selection (McGranahan and Swanton, 2017). As a consequence, tumors arising from within IEN are genetically distinct (Nakashima et al., 1995; Shinmura et al., 1998). Hence, Darwinian evolutionary clonal selection determines the loss of clones in field cancerisation and determines the size of multiple clones within the emerging SCC.

## HPV Infection and Stem Cell Expansion

Human papillomavirus (HPV) binds epithelial cell heparan sulfate proteoglycans and cell specific receptors to gain entry by both clathrin-dependent and -independent endocytosis (McMillan et al., 1999; Day et al., 2003; Shafti-Keramat et al., 2003; Spoden et al., 2008; Schelhaas et al., 2012; Day and Schelhaas, 2014). Infection leads to the establishment of the HPV circular double-stranded genome as a stable episome within some cells of the basal layer (Dell et al., 2003). In the case of alpha-HPV, the viral genome can integrate into the host genome, whereas for beta-HPV, the viral genome remains episomal (Quint et al., 2015). Viral replication proteins E1 and E2 are required for the maintenance of the viral genome in the basal layer (Frattini et al., 1996; Stubenrauch et al., 1998; McBride, 2013). HPV infection of epithelial basal cells may be non-selective and by chance may involve adult tissue stem cells that reside in this layer.

Most HPV infections are spontaneously cleared. For example, the risk of α-HPV female genital infection over a lifetime is up to 80% (Syrjanen et al., 1990), but within 1–2 years most individuals clear the virus (Rodríguez et al., 2008). Although HPV may not specifically bind epithelial adult tissue stem cells for infection, as discussed earlier, persistent and or latent infection is presumed to involve epithelial adult tissue stem cells, but has not been determined for all tissues as stem cell markers are lacking (Schmitt et al., 1996; Boxman et al., 1997; Maglennon et al., 2011; Kranjec and Doorbar, 2016). Notably, the proposed reservoir for latent β-HPV infection has been the hair follicles (Boxman et al., 1997; De Koning et al., 2007; Galloway and Laimins, 2015; Quint et al., 2015; Hufbauer and Akgül, 2017; Tommasino, 2017). Animal models substantiated these clinical findings and moreover showed that the sub-populations of infected hair follicle cells have increased clonogenic potential, a hallmark of adult tissue stem cells (Schmitt et al., 1996; Lanfredini et al., 2017). In the oncogenic β-HPV8 transgenic mouse model we observed skin thickening (acanthosis), which was evident from birth and attributable to an expansion of the Lrig1 hair follicle adult tissue stem population (Lanfredini et al., 2017). In the absence of overt cutaneous lesions, such as papilloma or carcinoma, both unsorted and Lrig1+ keratinocytes demonstrated increased colony forming efficiency (increased clonogenicity) consistent with an expansion in keratinocyte stem cell numbers. Similarly, earlier studies on the cottontail rabbit model of HPV infection had also demonstrated the hair follicle to be the site of persistent HPV infection and, through similar colony forming assays, an expansion of the hair follicle junctional zone keratinocyte stem cells was reported (Schmitt et al., 1996). In these two studies, papilloma arose as a result of continued keratinocyte stem cell expansion into the adjacent overlying epidermis. For example, human benign cutaneous viral warts similarly result from keratinocyte stem cells expansion (Egawa, 2003). It is possible that the immune privilege provided by the stem cells niche prevents immune attack, thereby facilitating long term infection.

## HPV-Induced Epidermal Proliferation

In cervical lesions caused by the α-HPVs, the viral oncogenes E6 and E7 increase proliferation of suprabasal epithelial cells. Along with E1 and E2, viral replication requires E6 and E7 for entry into S-phase. Upon leaving the basal layer, keratinocytes enter into a program of terminal differentiation in order to produce a protective barrier. However, in HPV infection, suprabasal cells continue to proliferate and are prevented from entering terminal differentiation (Sherman et al., 1997; Doorbar, 2006). Oncogenic viruses, including HPV, deregulate cell growth by disruption of pRb (retinoblastoma protein) binding to the E2F family of transcription factors though E7 binding pRb. Host p21 and p27 cyclin-dependent kinase inhibitors moderate the ability of E7 to drive cell proliferation in some cells (Doorbar, 2006; Tomaić, 2016). Inactive complexes with E7 and cyclin E occur within differentiating keratinocytes that express high levels of p21 and p27 (Noya et al., 2001; Akgül et al., 2006). In synchrony, high-risk α-HPV E6 prevent growth arrest or apoptosis by binding to p53, thus leading to p53 ubiquitination and degradation. In benign infections, proliferating cells remain in the epithelial basal layer, including within the hair follicle. As the infected cell enters the suprabasal cell layers of the epidermis, virus production is switched on resulting in viron assembly (Peh et al., 2002).

## HPV Infection Induces Stem Cell Expansion and Self-Renewal Pathways

Fluorescent labeling studies in mice using lineage tracing have concluded that stem cell division is prominently (∼90%) asymmetric; in which there is renewal of the stem cell and a daughter cell that is committed to terminal differentiation (Clayton et al., 2007; Doupé et al., 2012). Stochastic cell division in basal cells, including stem cells, can lead to HPV infection clearance. Mathematical modeling together with epidemiological data suggests that natural stem cell dynamics contributes >80% toward viral clearance rather than rejection by the immune system (Ryser et al., 2015). Thus, factors that promote adult tissue stem cell symmetrical cell division resulting in an increase in stem cell numbers may perpetuate infection accounting for the correlation between the increased risk of persistent infections associated cervical cancer and long-term use of combined oral contraceptives (Muñoz et al., 2006). This may also explain the basis for why trauma, ultraviolet light and repetitive exposure to the virus are essential in maintaining site-specific HPV infection (Kranjec and Doorbar, 2016).

Adult tissue stem cell expansion, as proposed for the mechanism of HPV-induced field cancerisation, is dependent on symmetrical division of existing stem cells. As discussed, HPV viral oncogenes will drive proliferation of infected adult tissue stem cells by targeting p53 or pRb. Importantly, the binding of E7 to pRb releases repression of both sex determining region Y-box 2 (Sox2) and octamer-binding transcription factor 4 (Oct4) (Kareta et al., 2015). Similarly, α-HPV E6 mediated degradation of p53 results increased Nanog expression, which is normally transcriptionally repressed by p53 (Lin et al., 2005). Thus, HPV early region genes promote self-renewal pathways.

In addition, high-risk α-HPV E7 induces expression of the key transcription factor Oct4 and also directly to enhance activation of its target genes (Brehm et al., 1997, 1999; Organista-Nava et al., 2016). Another key transcription factor, Kruppel-like factor 4 (Klf4), is also upregulated and hypoSUMOylated by high risk α-HPV E6 (Gunasekharan et al., 2016). Simultaneously, β-HPV E6 blocks differentiation by inhibition of C/EBPα, Notch signaling and Hes1 upregulation (Tyagi et al., 2016; Kranjec et al., 2017; Marthaler et al., 2017; Meyers et al., 2017). β-HPV E6 specifically binds to a cellular target MAML1, resulting in the inhibition of Notch-mediated transcription, which is important to keep infected keratinocytes in a proliferative state (Meyers et al., 2017). α-HPV E7 also prevents histone3 Lysine27 (H3K27) trimethylation and therefore maintains adult tissue stem cells in a permissive epigenetic state (McLaughlin-Drubin et al., 2011). Thus, HPV causes proliferation of adult tissue stem cells and maintains stemness of these cells as they egress from the stem cell niche, consistent with expression of stem cell proteins and observations *in vitro* of increased colony forming efficiency (Hufbauer et al., 2013; Lindquist et al., 2014).

## Transition From HPV-Induced Stem Cell Expansion to IEN

The earliest evolution of HPV-induced stem cell expansion into visible lesions is the presence of dysregulated stratification within the epidermis, resulting in benign keratoses (the archetypal lesion in epidermodysplasia verruciformis) or cutaneous warts. Similarly, mucosal HPV lesions include condyloma or leukoplakia within the genitalia and oral mucosa (Cubie, 2013). In addition, persistent infections with high-risk HPV types simultaneously trigger neoplastic change (Rodríguez et al., 2010).

The transition from benign to premalignant lesion has been characterized by TP53 immunostaining, resulting from mutation acquisition, and manifesting as a small micro-clonal expansion comprising of 60–3000 cells presenting clinically as an actinic (solar) keratosis or leukoplakia (Jonasson et al., 1996; Ren et al., 1966; Ponten et al., 1997; Waridel et al., 1997; Garcia et al., 1999; van Houten et al., 2002). In the skin, these p53 micro-clonal patches were larger and more frequent in sun-exposed than sun-shielded areas, suggesting that mutations arise from UV. In addition, HPV is able to inhibit DNA repair through E6 protein expression, facilitating acquisition of p53 mutations (Wallace et al., 2012; Hufbauer et al., 2015; McKinney et al., 2015). Gain-of-function p53 mutation acquisition results in persistence of the protein within cells to promote transformation (Caulin et al., 2007).

Progression of field cancerisation toward severe IEN is associated with loss of the viral episome. In HPV infection, such as benign warts, epithelial proliferating cells remain in the basal layers, with genome amplification and virion assembly occurring within the suprabasal cell layers (Peh et al., 2002; Middleton et al., 2003). In the case of the high-risk HPV types the relative thickness of the basal layers is increased, presumably due to expansion in the number of adult tissue stem cells. Progression to IEN is characterized by a loss of terminal differentiation and therefore the expression of viral coat proteins is retarded (**Figure 2**) (Middleton et al., 2003). For example in cervical IEN, increasing dysplasia is associated with reduced virion production and loss of viral episomes. This phenomenon is even more evident in the case of skin infection by β-HPV types, which do not integrate into the host genome, and do not maintain viral DNA in the late stages of skin cancer progression. For example, SCC that develop within HPV associated Organ Transplant Recipient (OTR) field cancerisation no longer express β-HPV proteins (Borgogna et al., 2014) Similarly, HPV expression was lost during actinic keratosis transformation to SCC in a nude mouse xenograft model (Borgogna et al., 2018). Hence, the progression to cancer from IEN occurs independent of virus production, and for the beta genotypes in the skin, this is referred to as the “hit and run” mechanism of carcinogenesis (Howley and Pfister, 2015; Quint et al., 2015).

Field cancerisation emerging from HPV induced amplification of adult tissue stem cells results from additional environmental induced mutations. The area of IEN can be large, in the oral cavity it can be over 7 cm in diameter and is predisposed to multiple primary HNSCC and therefore poor prognosis (Tabor et al., 2002, 2004; Baxi et al., 2014). Intriguingly, HPV associated HNSCC demonstrate a favorable response to chemotherapy (Hayes et al., 2015; Vokes et al., 2015). Likewise, HPV and non-HPV vulvar SCC have distinct mutational profiles and moreover multiple primaries developing from within HPV IEN demonstrate separate clonal basis (Rosenthal et al., 2002; Hampl et al., 2007). Hence, HPV-induced adult tissue stem cell expansion risks the generation of IEN that in turn is predisposed to further transformation resulting in multiple primary tumors.

## HPV Infection Driven Cancer Stem Cells

Many cancers exhibit hierarchical growth with evidence of differentiation consistent with the cancer stem cell model (Colmont et al., 2012). Wherein a subset of cancer cells, called “cancer stem cells”, which continue to exhibit stem cell characteristic, serve to promote tumor growth through self-renewal with symmetric and asymmetric cell division (Patel et al., 2012; Colmont et al., 2013). There is evidence of active self-renewal in HPV-induced female genital tract cancers, cervical and vulval cancers, which characteristically express the stem cell transcription factors Sox 2, Oct4, and Hes1 (Brustmann and Brunner, 2013; Kim et al., 2015; Napoletano et al., 2016; Gut et al., 2018). In cervical cancer, HPV gene E6 can enhance self-renewal associated hedgehog transcription factor Gli1 expression and therefore increase cancer stem cell numbers (Vishnoi et al., 2016).

Head and neck squamous cell carcinoma (HNSCC) identification and characterisation of cancer stem cells has been supported by *in vitro* and *in vivo* assays (Prince et al., 2007). Similar to HPV-induced female genital tract cancers, the self-renewal associated transcription factor Sox2 was found expressed in HPV associated HNSCC, resulting from HPV E6/7 associated PI3K-AKT pathway activation (Keysar et al., 2016; Xi et al., 2016). The ensuing HPV-associated HNSCC retain cancer stem cell markers, CD44, CD24, ALHD1, and functional side population characteristics (Tang et al., 2013; Lindquist et al., 2014; Pullos et al., 2015). Overall, HPV associated HNSCC has favorable outcome compared to non-HPV associated HNSCC, and intriguingly this has been attributed to reduced cancer stem cell frequency in HPV HNSCC (Rietbergen et al., 2014; Vlashi et al., 2016). High numbers of cancer stem cells in HNSCC, irrespective of HPV status, is associated with poor outcome and lack of response to both radiotherapy and chemotherapy (Linge et al., 2016; Modur et al., 2016). Hence, the role of HPV to cause both normal stem cell and cancer stem cell expansion, may establish the basis for cancer stem cell driven tumor growth and influence cancer outcome.

## Conclusion

This review has focused on HPV infection, notably oncogenic genotypes from both the alpha and beta genus. Within the tropic tissue that was breached to allow viral entry, persistent infection requires that resident adult tissue stem cells are infected. HPV-infected adult tissue stem cells, similar to other HPV-infected cells are forced to proliferate, leading to their expansion as adult tissue stem cells beyond their native niche. This expansion renders them susceptible to environmental carcinogens. In the case of skin, β-HPV genotypes induce hair follicle junctional zone keratinocyte stem cells to proliferate and expand into the overlying epidermis, whereupon they are susceptible to UV-induced mutations. Transformational mutations result in field cancerisation, with additional driver mutations, causing clonal selection as IEN progresses from mild to severe. Additional mutations then can give rise to multiple cancers. Hence, HPV-induced stem cell expansion predisposes to and, through viral oncogene expression, induces the generation of cancer stem cells, which in turn can define the fate of tumor and patient prognosis. Hence, we propose that the ability of oncogenic HPV infection to manipulate adult tissue stem cells underpin its ability to drive cancer growth through promotion of cancer stem cells.

## Author Contributions

CO, SL, and GP conceived the idea. CO, SL, CB, MG, and GP drafted the manuscript with inputs from all authors. All authors have made final approval for the final version to be submitted.

## Conflict of Interest Statement

The authors declare that the research was conducted in the absence of any commercial or financial relationships that could be construed as a potential conflict of interest.

## Footnotes

1 http://www.cancerresearchuk.org/health-professional/cancer-statistics/incidence/common-cancers-compared
